# CaMKII Neurons in the Dentate Gyrus Are Involved in Regulating Cognitive Impairment in Mice Induced by Stress Caused by Violence

**DOI:** 10.3390/ijms27010226

**Published:** 2025-12-25

**Authors:** Gaojie Shao, Dan Liu, Zijun Liu, Qian Xiao, Qing Shang, Hongyan Qian, Jie Tu, Xinshe Liu

**Affiliations:** 1 College of Forensic Medicine, Western China Science and Technology Innovation Harbor, Xi’an Jiaotong University, Xi’an 710061, China; sgj0312@stu.xjtu.edu.cn (G.S.);; 2 CAS Key Laboratory of Brain Connectome and Manipulation, The Brain Cognition and Brain Disease Institute (BCBDI), Shenzhen Institute of Advanced Technology, Chinese Academy of Sciences, Shenzhen 518055, China; liudan@siat.ac.cn (D.L.);; 3 Department of Forensic Medicine, School of Basic Medical Science, Wenzhou Medical University, Wenzhou 325035, China

**Keywords:** 3D behavior atlas, stress caused by violence, post-stress cognitive impairment

## Abstract

Post-stress cognitive impairment (PSCI) is defined as a persistent neuropsychiatric condition characterized by deficits in memory consolidation, executive functioning, and environmental interaction following exposure to violent stress. Despite its high incidence, PSCI remains underdiagnosed and lacks effective therapeutic strategies, posing a substantial societal burden and highlighting a critical gap in neuropsychiatric research. A major constraint in mechanistic studies is the persistent reliance on conventional paradigms, notably the Y-maze and novel object recognition test. Their limited sensitivity and poor translational relevance to human cognitive dysfunction, compounded by slow methodological innovation, significantly impede progress. Furthermore, the specific brain regions or neuronal populations contributing to PSCI pathogenesis are insufficiently explored. To address this, we assessed post-stress cognitive impairment in mice using a triple approach: Skinner box assays, traditional behavioral paradigms, and integrated 3D ethological analysis. This multi-method framework provides novel insights for refining animal models and advancing mechanistic understanding. Using c-Fos-based whole-brain screening, we identified the dentate gyrus (DG) as a key region involved in PSCI. Stress caused by violence markedly increased activity in DG CaMKII-expressing neurons. Chemogenetic inhibition of these neurons effectively alleviated stress-induced mild cognitive impairment phenotypes. In summary, by applying novel behavioral assessment tools, this study demonstrates that DG CaMKII neurons play a critical role in regulating post-stress cognitive impairment.

## 1. Introduction

Stress caused by violence is a growing global health concern. According to the World Health Organization, its incidence has risen steadily since 2014 [1], affecting millions worldwide. Acute stress caused by violence (ASCV) encompasses a broad spectrum of traumatic experiences, including physical and sexual assault, psychological aggression [2], verbal abuse, and witnessing violence [3,4,5,6,7]. Beyond immediate physical harm—such as fractures, lacerations, and chronic pain—exposure to stress caused by violence induces long-lasting neuropsychiatric sequelae, including PSCI, complex post-traumatic stress disorder, and acute stress disorder [8]. These conditions often manifest as memory loss, executive dysfunction, depression, anxiety, and increased suicide risk [2]. Among them, PSCI imposes a particularly heavy burden on individual cognition and social functioning, yet remains significantly underdiagnosed, poorly understood, and lacking established therapeutic targets.

Cognitive impairment involves deficits in higher-order brain functions, including declines in memory formation, learning, reasoning, and information integration [9]. Several hypotheses have been proposed, such as structural brain injury [10,11,12], psychogenic factors [13,14], dysregulation of neuroendocrine pathways including the brain–gut axis [15], and neuroinflammatory cascades driven by iron deposition [16]. However, mechanistic understanding remains limited, partly due to challenges in accurately modeling PSCI and quantifying its behavioral correlates in animals.

To address these challenges, this study established a refined behavioral evaluation framework based on the Behavior Atlas platform [17]—a high-dimensional profiling system that integrates automated 3D motion capture with machine learning. Unlike conventional paradigms that assess isolated behavioral dimensions, this system enables continuous, multidimensional tracking of learning, memory, exploration, and decision-making.

Using this platform, we systematically evaluated cognitive performance in rodents exposed to stress caused by violence by combining operant learning paradigms with 3D ethological tracking, thereby detecting subtle behavioral alterations beyond the limits of conventional assays. To link behavior with underlying neural activity, we performed whole-brain c-Fos mapping, which identified stress-responsive regions implicated in PSCI. Stress caused by violence markedly increased the activity of CaMKII-expressing neurons in the DG. Chemogenetic inhibition of these neurons alleviated stress-induced mild cognitive impairment, suggesting that hippocampal circuitry plays a central role in mediating stress-induced learning and memory deficits.

Moreover, because cognitive impairment following stress caused by violence is often overlooked in forensic and clinical settings, the successful establishment of this acute stress–induced murine model provides a valuable platform for translational research. Together, the development of advanced behavioral phenotyping methods and the identification of hippocampal mechanisms underlying PSCI represent a significant step toward elucidating its pathophysiology and guiding future therapies.

## 2. Results

### 2.1. ASCV Impairs Spatial Memory in Mice

**Figure 1:**
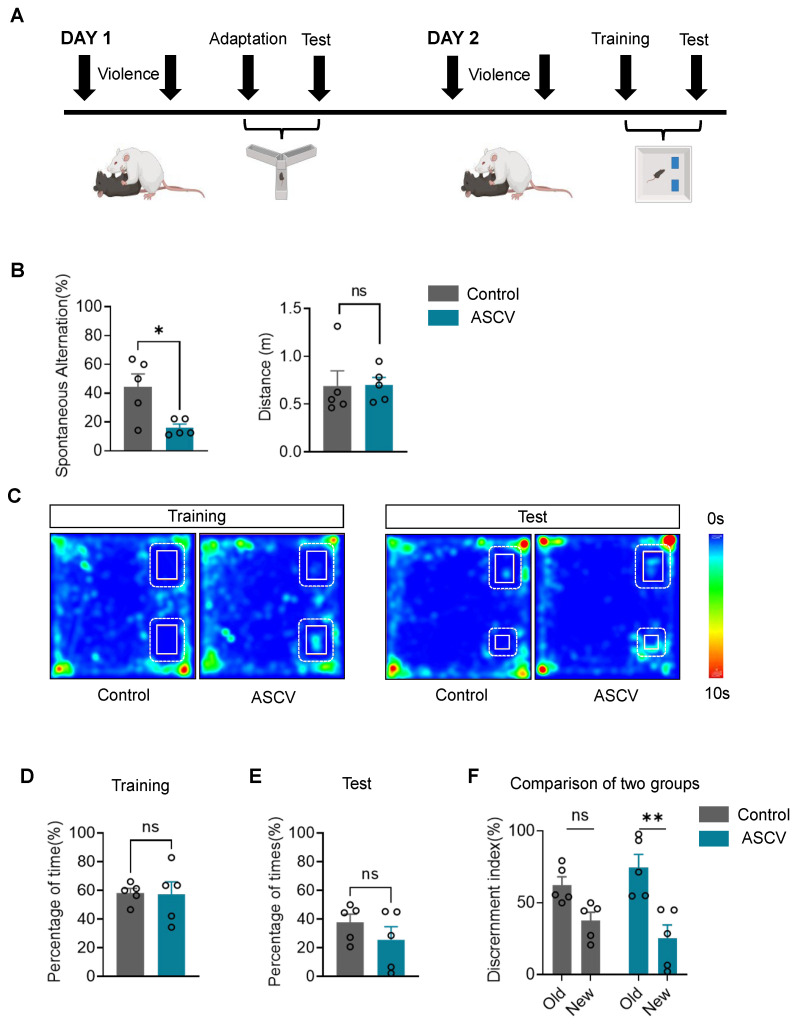
ASCV impairs spatial memory in mice. (**A**) Schematic and timeline of ASCV modeling, novel object recognition test (NORT) and Y-maze testing. (**B**) Y-maze performance: Reduced spontaneous alternation rate in ASCV-exposed mice (unpaired *t*-test: *t* = 2.967, *p* = 0.018) versus controls, with no difference in locomotion (distance traveled in novel arms: *t* = 0.054, *p* = 0.958). (**C**) NORT heatmaps of object exploration. (**D**,**E**) NORT behavioral metrics: Comparable exploration times for two identical objects during training (*t* = 0.088, *p* = 0.932); Non-significant novelty preference in test phase (*t* = 1.121, *p* = 0.295); (**F**) Significant reduction in novel object discrimination for ASCV-exposed mice (Control: *t* = 2.289, *p* = 0.062; ASCV: *t* = 3.778, *p* = 0.005). (n = 5/group). Data analyzed via unpaired *t*-tests; presented as mean ± SEM. ns, not significantly different. * *p* < 0.05, ** *p* < 0.01.

To investigate the cognitive consequences of stress caused by violence, a validated ASCV model was applied to induce spatial memory deficits in C57BL/6J mice. Spatial memory alterations were assessed 30 min post-exposure using the Y-maze and novel object recognition test (Figure 1A).

In the Y-maze, mice subjected to ASCV exhibited a 36.4% reduction in spontaneous alternation accuracy compared to controls (Figure 1B), indicating impaired working memory. Total distance traveled did not differ between groups, ruling out confounding effects of locomotor activity. Compared to controls, mice in the ASCV group showed less exploration towards the novel open arms, as visualized in Y-maze heatmaps (Supplementary Figure S1E).

During the novel object recognition training phase, both groups showed comparable exploration of the two identical objects (Figure 1C,D), confirming no inherent object bias. During the retention test conducted 2 h post-training, although no significant group difference emerged in time spent exploring the novel object, ASCV mice displayed a clear reduction in novelty preference (Figure 1E). This trend was substantiated by a 34% decline in the discrimination index in the ASCV group relative to controls (Figure 1F), reinforcing spatial memory impairment.

Collectively, these findings demonstrate that exposure to ASCV disrupts spatial memory, as evidenced by reduced alternation accuracy and impaired object discrimination, while sparing baseline locomotor capacity.

### 2.2. ASCV Impairs Spatial Memory and Exploratory Behavior in Mice

**Figure 2:**
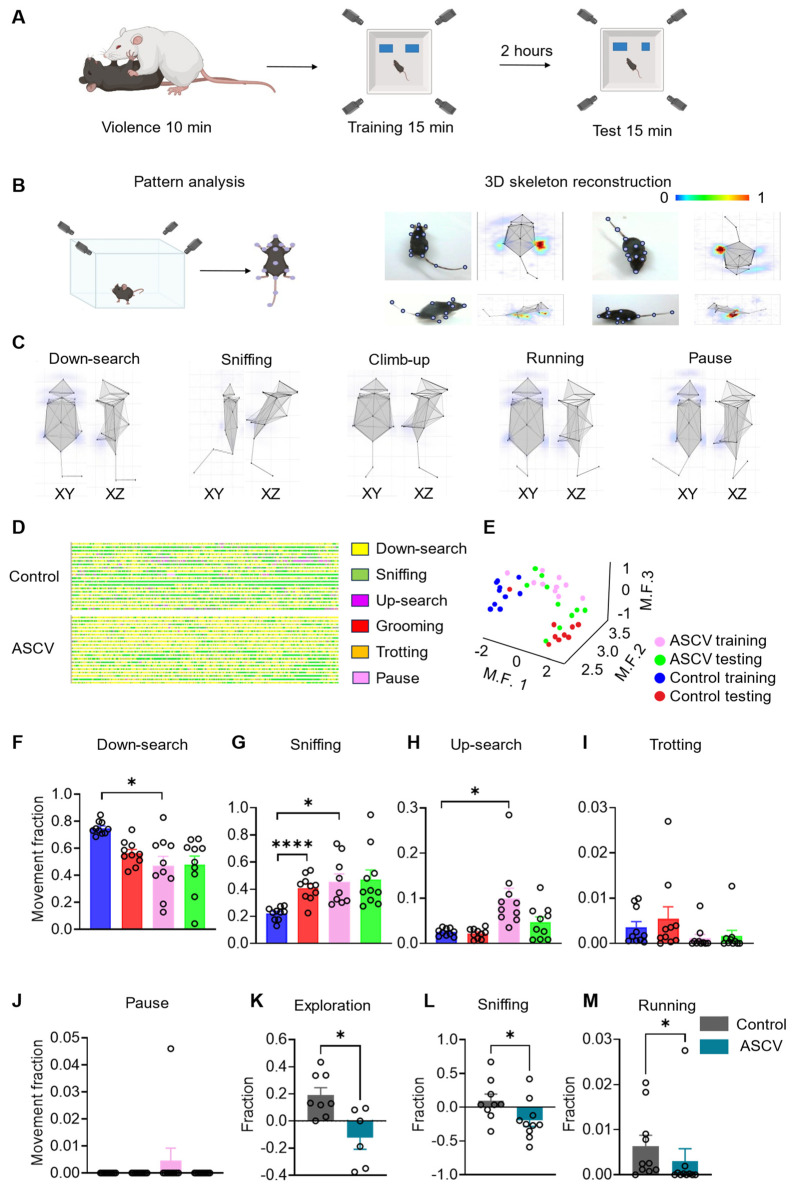
Integrated 3D ethological profiling with novel object recognition reveals exploratory deficits induced by ASCV. (**A**) Schematic and timeline of ASCV modeling combined with 3D ethological analysis and novel object recognition. Phases: Training and Testing. (**B**) Three-dimensional tracking system and annotated anatomical landmarks for fine-grained motion analysis. (**C**) Three-dimensional skeletal reconstructions (XY- and XZ-planes) of six representative behavioral subclasses. (**D**) Action spectrograms of five behavioral subclasses in Control (CON) and ASCV groups during integrated 3D-object recognition tasks. (**E**) Dimensionality-reduced clustering plots (training vs. testing phases) of behavioral parameters for CON and ASCV-exposed mice. (**F**–**J**) Histograms quantifying behavioral subclasses: Down-search: (one-way ANOVA, F = 8.698, *p* = 0.0057), Multiple comparisons: CON-Training vs. ASCV-Training (*p* = 0.0178). Sniffing: (one-way ANOVA, F = 7.427, *p* = 0.0056), Multiple comparisons: CON-Training vs. CON-Test (*p* < 0.0001); CON-Training vs. ASCV-Training (*p* = 0.0304). Climbing: (one-way ANOVA, F = 8.093, *p* = 0.0065), Multiple comparisons: CON-Training vs. ASCV-Training (*p* = 0.0392). (**K**,**L**) Discrimination indices for novel vs. familiar objects based on exploration clusters (*t* = 2.865, *p* = 0.0457) and sniffing subclasses (*t* = 2.146, *p* = 0.0466). (**M**) Reduced running behavior during total object exploration in ASCV-exposed mice (Mann–Whitney test: *p* = 0.0186). (n = 10/group). Data analyzed via unpaired *t*-tests; presented as mean ± SEM. * *p* < 0.05, **** *p* < 0.0001.

To examine the impact of stress caused by violence on cognitive and behavioral function, a validated ASCV model was applied to C57BL/6J mice. At 30 min post-ASCV exposure, spatial memory and exploratory behavior were assessed using a 3D behavioral analysis system integrated with the novel object recognition task (Figure 2A).

The novel object recognition apparatus was positioned within a sound-attenuated behavioral chamber equipped with four ceiling-mounted cameras to enable synchronized, multi-angle acquisition of high-resolution video data. Mice were introduced into the center of the open-field arena (Figure 2B), and 16 anatomical landmarks, including nose, right/left ears, neck, back, anterior/middle/posterior sections of tail, right/left forelimbs, right/left paws, right/left hindlimbs, and right/left feet, were manually annotated across four anatomical planes (Figure 2B). These landmark coordinates were processed via 3D ethological software (Behavior Atlas Analyzer, Version 1.2.3) to reconstruct a detailed skeletal model and generate corresponding heatmaps (Figure 2B), enabling granular motion analysis.

All recorded behaviors were classified into 40 discrete action modules by the 3D behavioral analysis platform and subsequently classified into four functional clusters: exploration (down-search, sniffing, jumping, climbing, rearing, up-search, falling), locomotion (running, trotting, left/right turning, stepping), maintenance (grooming), and inactive (pausing). Dominant behaviors during the novel object recognition task included down-search, sniffing, up-search, running, and pausing, each visualized through skeletal trajectories in XY- and XZ-planes (Figure 2C).

To further delineate behavioral phenotypes, action spectrograms and dimensionality-reduced clustering plots were constructed across both training and testing phases (Figure 2D,E). Mice exposed to ASCV demonstrated reduced exploratory complexity and behavioral diversity, as reflected by compressed action spectrograms (Figure 2D) and attenuated cluster separability (Figure 2E), suggesting impaired behavioral discrimination and flexibility.

Object-centered spatial partitioning enabled precise quantification of exploratory behaviors. While the proportion of trotting and pausing remained comparable between groups (Figure 2I,J), indicating preserved motor function, ASCV mice showed a significant increase in up-search activity during the training phase that normalized during testing (Figure 2H), suggesting accelerated retention interval of spatial memory traces following exposure to violence. Compared to controls, animals in the ASCV group exhibited significantly reduced exploratory behavior (down-search) across both training phases (Figure 2F), reflecting stress-induced impairment in environmental engagement. Sniffing behavior, particularly sensitive to novelty-driven attention, increased during testing in control mice but failed to increase in ASCV-exposed mice, indicating impaired novelty discrimination. Although mice subjected to ASCV initially exhibited heightened sniffing during training, this response declined during testing (Figure 2G), consistent with a failure in memory consolidation rather than sensory encoding.

Discrimination indices computed from both exploratory clusters (Figure 2K) and sniffing subclasses (Figure 2L) revealed significant deficits in novel object recognition in ASCV-exposed mice relative to controls. Additionally, total object exploration time, primarily driven by running behavior, was significantly reduced in the ASCV group (Figure 2M). These results demonstrate that ASCV impairs both exploratory engagement and spatial memory retention in mice.

Post hoc analyses confirmed that ASCV exposure did not impair gross motor function but significantly disrupted down-search activity—a hallmark of impaired exploratory behavior. While overall sniffing time remained comparable across phases, the absence of novelty-driven elevation in this behavior during testing indicated disrupted spatial memory consolidation. Critically, mice in the ASCV condition showed diminished discrimination indices for novel objects across multiple behavioral subclasses, including exploration, sniffing, and locomotion, thus demonstrating that acute stress caused by violence compromises both spatial memory retention and fine-grained environmental interactions.

### 2.3. ASCV Induces Deficits in Operant Learning and Working Memory in Mice

**Figure 3:**
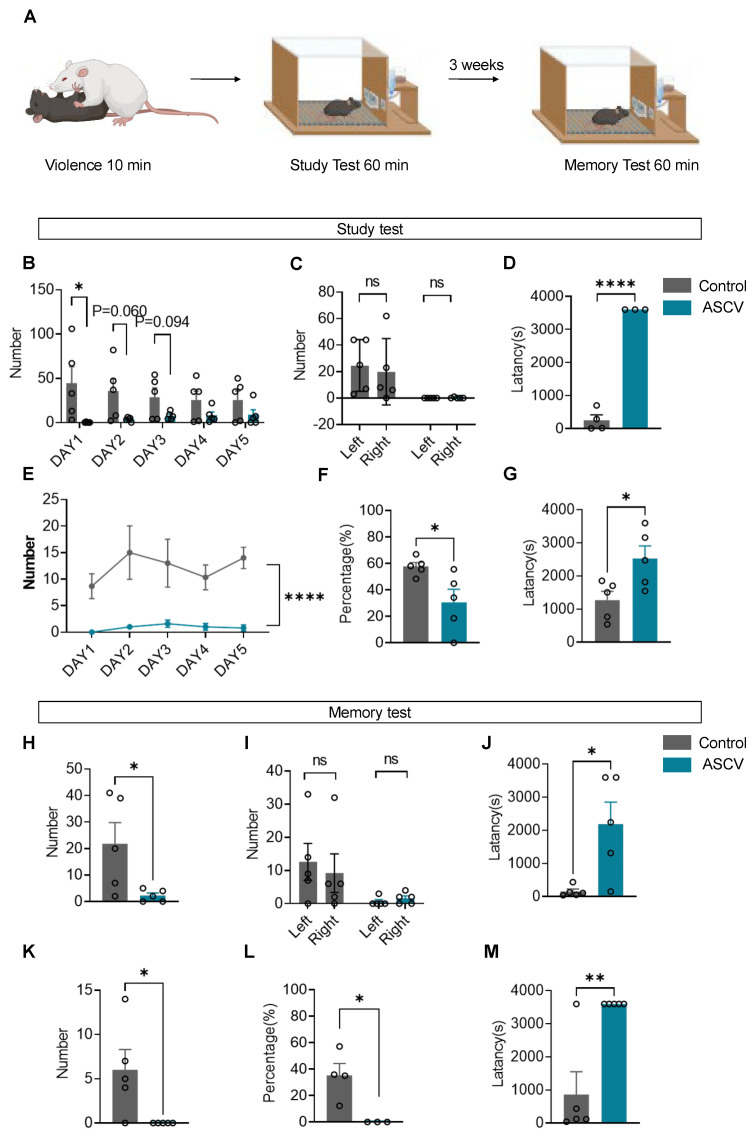
Skinner Box operant conditioning reveals learning and memory deficits induced by ASCV. (**A**) Schematic and timeline of ASCV modeling and Skinner box lever-pressing tasks. Phases: Study and Memory tests. (**B**,**E**–**G**) Learning phase metrics over 5 days: Total lever presses (unpaired *t*-test: *t* = 2.340, *p* = 0.0474), Effective lever presses (two-way ANOVA: F = 92.10, *p* < 0.0001), Task accuracy (*t* = 2.645, *p* = 0.0295), Effective lever-press latency (*t* = 2.659, *p* = 0.0289). (**C**) Day 1 lever-press counts (left vs. right) during learning: Control: *t* = 1.120, *p* = 0.2952 (unpaired *t*-test); *p* = 0.6825 (Kolmogorov-Smirnov test), ASCV: *p* > 0.9999 (Kolmogorov–Smirnov test). (**D**) Day 1 lever-press latency (*t* = 17.40, *p* < 0.0001). (**H**–**M**) Memory retrieval phase metrics: Total lever presses (*t* = 2.431, *p* = 0.0411), Lever preference (Control: *p* = 0.3571; ASCV: *p* > 0.9999, Kolmogorov–Smirnov test), Total latency (*t* = 3.024, *p* = 0.0165), Effective lever presses (*t* = 2.606, *p* = 0.0313), Task accuracy (*t* = 3.228, *p* = 0.0233), Effective latency (*t* = 3.981, *p* = 0.0041). (n = 5/group). Data analyzed via unpaired *t*-tests; presented as mean ± SEM. ns, not significantly different. * *p* < 0.05, ** *p* < 0.01, **** *p* < 0.0001.

Cognitive impairments following ASCV were evaluated using a Skinner box paradigm designed to assess operant learning and working memory in C57BL/6J mice. At 30 min post-ASCV exposure, mice underwent an FR-1 lever-pressing task paired with a 10% sucrose reward (Figure 3A).

During the initial acquisition phase, mice exposed to ASCV demonstrated a significant reduction in total lever presses on Day 1 compared to controls (Figure 3B), although this disparity narrowed with extended training. Analysis of left/right lever preferences revealed no inherent lateral bias in either group on Day 1 (Figure 3C). However, ASCV-exposed mice exhibited markedly prolonged lever-press latencies relative to controls (Figure 3D). Over the 5-day training period, stressed mice consistently produced fewer effective lever presses—defined as left-lever responses yielding reinforcement—compared to controls (Figure 3E). Additionally, the ASCV group displayed lower task accuracy (percentage of correct left-lever presses, Figure 3F) and increased effective latency (time to successful presses, Figure 3G), confirming deficits in operant learning and associative processing.

To assess retention of learned behavior, mice were returned to their home cages for a 3-week retention interval period, followed by a single-session working memory recall test. While lever preference remained equivalent across groups (Figure 3I), ASCV-exposed mice exhibited substantial reductions in total lever presses (Figure 3H), effective lever responses (Figure 3K), and task accuracy (Figure 3L). These impairments were accompanied by significant increases in both overall response latency (Figure 3J) and effective latency (Figure 3M), indicating a pronounced decline in working memory function.

Collectively, these findings demonstrate that ASCV significantly impairs both the acquisition and retention of operant behavior, as evidenced by reduced reinforcement-driven responding, impaired task accuracy, and prolonged decision latency. The ASCV model thus reveals potent disruption of hippocampal- and prefrontal cortex-mediated cognitive processes, implicating acute trauma as a key driver of learning and memory dysfunction.

### 2.4. DG Responds to Acute Stress Caused by Violence

**Figure 4:**
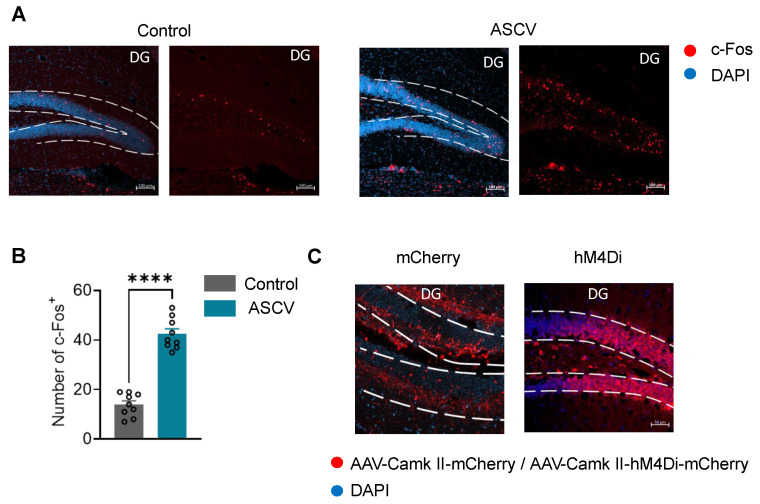
DG Responds to PSCI and Viral Injection in the DG. (**A**) Representative images of c-Fos expression in the DG of mice from both groups after PSCI. Scale bar: 100 μm. (**B**) Quantitative data of c-Fos in the DG of mice from both groups after PSCI (unpaired *t*-test: *t* = 11.00, *p* < 0.0001) (N = 9/group). (**C**) Representative confocal images showing viral injection in the DG. Scale bar: 50 μm. Data were analyzed via independent sample *t*-test and are presented as mean ± SEM. ****, *p* < 0.0001.

To further explore the brain regions involved in causing this phenomenon, we performed perfusion and sectioning on mice that had undergone acute stress caused by violence and conducted c-Fos screening of the whole brain. Among the results, the c-Fos increased expression in the DG was particularly significant (Figure 4A,B).

We performed whole-brain c-Fos screening following ASCV exposure. While other regions (including PVN, LH, and PH) showed some differential activation, the dentate gyrus (DG) of the hippocampus exhibited the most pronounced and consistent c-Fos increase. Given the hippocampus’s established central role in learning and memory, we prioritized the DG for further mechanistic investigation (Supplementary Figure S1D).

According to existing literature reports, Ca^2+^-calmodulin-dependent kinase II (CaMKII) contributes to synaptic transmission and is extremely abundant in the brain. It significantly mediates hippocampal long-term potentiation (LTP), a form of synaptic plasticity considered essential for learning and memory. However, CaMKII also mediates LTP impairments associated with Alzheimer’s disease (AD) and global cerebral ischemia (GCI)—two distinct conditions both linked to learning and memory deficits. In both cases, CaMKII inhibitors prevented these LTP impairments [18,19].

We also used fiber photometry calcium signaling recording to test the responses of CaMKII neurons in the DG to other negative stimuli. The results revealed that CaMKII neurons in the DG respond to negative stimuli, such as tail suspension (Supplementary Figure S1C), suggesting their potential involvement in the stress processing generated by such stimuli. This indicates that CaMKII neurons in DG may serve as a key regulatory target for brain cognitive dysfunction induced by various negative stimuli. Consequently, we have reason to suspect that CaMKII neurons in DG would also respond to stress caused by various negative stimuli (Supplementary Figure S1C).

Therefore, we injected the virus AAV-CaMKII-hM4Di-mCherry carrying inhibitory chemogenetic components and the control virus AAV-CaMKII-mCherry into the DG of mice, and examined the viral expression (Figure 4C), to investigate whether inhibiting the activity of excitatory neurons in the DG alters the mild cognitive impairment phenotype in mice subjected to the ASCV model.

### 2.5. Inhibition of Excitatory Neurons in the DG Alters the Mild Cognitive Impairment Phenotype in Mice Induced by Acute Stress Caused by Violence

**Figure 5:**
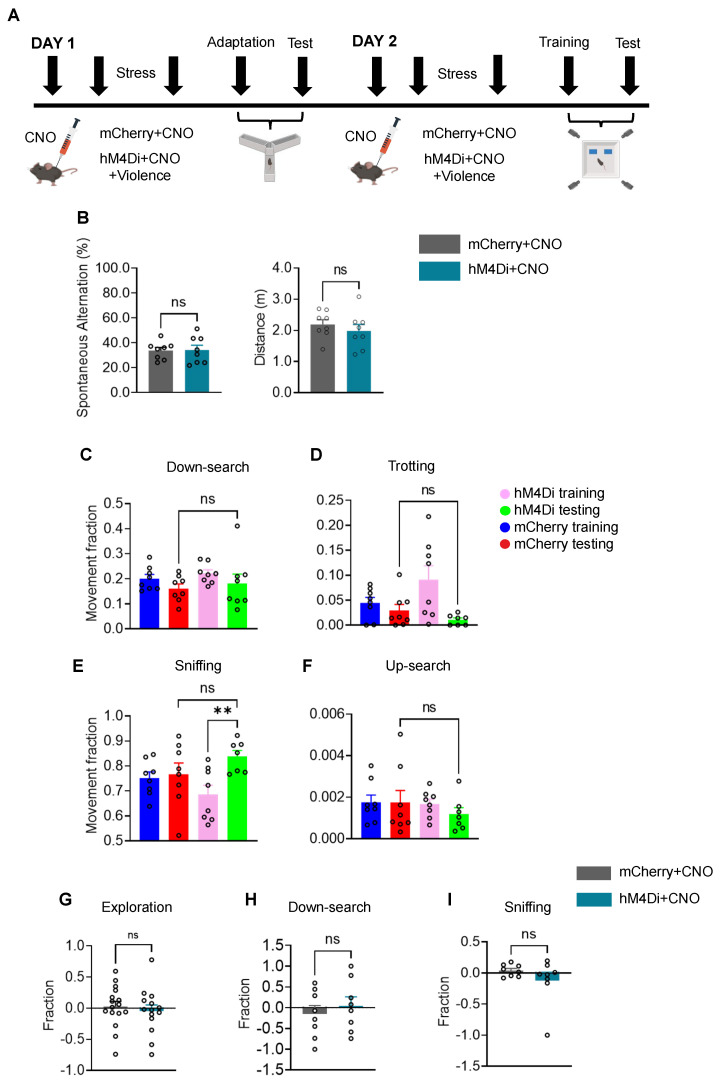
Inhibition of excitatory neurons in the DG alleviates spatial memory decline induced by the acute stress caused by the violence model. (**A**) Schematic diagram and timeline of CNO injection, acute stress caused by violence, Y-maze test and the novel object recognition test combined with 3D behavioral analysis. (**B**) Y-maze test: spontaneous alternation arm selection (unpaired *t*-test: *t* = 0.09537, *p* = 0.9254) and movement distance in the novel open arms (unpaired *t*-test: *t* = 0.7992, *p* = 0.4375). (**C**–**F**) Histograms of four sub-categories of behaviors after detailed behavioral annotation in the novel object recognition test combined with 3D behavioral analysis for mCherry and hM4Di mice (unpaired *t*-test: Down-search: *t* = 0.4898, *p* = 0.6319; Trotting: *t* = 1.398, *p* = 0.1856; Up-search: *t* = 0.8050, *p* = 0.4353; Sniffing: mCherry-Testing group vs. hM4Di-Testing group: *t* = 1.333, *p* = 0.2053; hM4Di-Training group vs. hM4Di-Testing group: *t* = 3.335, *p* = 0.0054). (**G**–**I**) Histograms of the discrimination index for novel vs. familiar objects in the novel object recognition test combined with 3D behavioral analysis after detailed behavioral annotation for CON and ASCV mice, including broad exploratory behavior (unpaired *t*-test: *t* = 0.5736, *p* = 0.5708), Down-search (unpaired *t*-test: *t* = 0.6534, *p* = 0.5241), and sniffing in place (unpaired *t*-test: *t* = 1.182, *p* = 0.2568) (N = 8/group). Data were analyzed via independent sample *t*-test and are presented as mean ± SEM. ns, not significant. **, *p* < 0.01.

CNO (1 mg/kg, i.p.) was administered 30 min prior to behavioral testing to achieve chemogenetic inhibition of CaMKII neurons in the DG. Thirty minutes later, the ASCV modeling was conducted. Thirty minutes after the completion of modeling, the Y-maze and a novel object recognition device combined with 3D behavioral analysis (3D-BA) (Figure 5A) were used to assess changes in spatial memory and exploratory ability in mice, respectively.

In the Y-maze test, compared with control mice, the hM4Di group showed no significant decrease in the correct rate of alternation arm choices and no significant difference in the movement distance in the two novel open arms (Figure 5B). This indicates that inhibiting excitatory neurons in the DG alleviates spatial memory impairment induced by acute stress caused by violence without affecting motor function in mice. Y-maze heatmap analysis demonstrated that chemogenetic inhibition of CaMKII neuronal activity in the DG restored exploration of the novel open arms in stressed mice to a level comparable to that of non-stressed controls (Supplementary Figure S1F).

Subsequently, we employed a novel object recognition test combined with 3D behavioral analysis. After detailed behavioral annotation and zone segmentation, statistical analysis of action segment proportions revealed no significant difference in trotting behavior between the two groups (Figure 5D), indicating that motor function was not impaired in ASCV model mice. No significant differences were observed between the two groups in up-search, down-search, or sniffing in place during the test phase. Moreover, the hM4Di group exhibited significantly increased sniffing in place toward the novel object compared to the familiar object (Figure 5C,E,F). These results suggest that inhibiting excitatory neurons in the DG alleviates spatial memory impairment induced by acute stress caused by violence without affecting motor function in mice.

We also quantified the spectrograms of each action segment in the novel object recognition test for both groups of mice (Supplementary Figure S1A), as well as scatter plots and bar charts showing the proportion of different types of action segments relative to the total actions during each phase of the novel object recognition test (Supplementary Figure S1B). These results further demonstrate that inhibiting the activity of CaMKII neurons in the DG effectively alleviates cognitive impairment induced by acute stress caused by violence in mice.

Finally, after zone segmentation and detailed action definition, we analyzed the discrimination index for novel objects in both groups. Whether expressed broadly as exploratory action categories or specifically as down-search or sniffing in place subcategories, no significant differences were observed between the two groups (Figure 5G–I). These results indicate that chemogenetic inhibition of DG excitatory neurons alters the phenotype of spatial memory impairment and novelty discrimination deficits induced by stress caused by acute violence, without affecting general motor function.

The above results demonstrate that after inhibiting excitatory neurons in the DG, ASCV did not alter motor function in either group, and the proportions of various actions, including down-search and sniffing in place, showed no significant differences between the two groups. This suggests that spatial memory ability in hM4Di group animals was improved after inhibiting excitatory neurons in the DG following ASCV modeling. No significant differences were found in the discrimination indices for novel versus familiar objects in broad exploratory action categories or specific subcategories such as sniffing in place and trotting, indicating that spatial memory and exploratory ability in the hM4Di group were enhanced after inhibiting excitatory neurons in the DG following ASCV modeling.

## 3. Discussion

This study advances current understanding of stress-induced cognitive dysfunction by establishing a high-resolution behavioral assessment framework that reveals acute deficits in spatial memory, learning acquisition, and memory retention following stress caused by violence. The findings have significant implications for translational neuroscience and preclinical modeling of stress-related cognitive disorders.

Consistent with prior studies [20,21,22], acute exposure to stress caused by violence significantly disrupted Y-maze alternation and novel object recognition in mice, indicating spatial memory. Beyond traditional measures, 3D ethological profiling detected fine-scale disruptions in exploratory behaviors—such as reduced novelty-driven sniffing and down-search activity—that are often missed by conventional metrics. Skinner box operant conditioning further revealed deficits in associative learning, shown by reduced accuracy, fewer effective lever presses, and longer latencies. Together, these results reaffirm the detrimental impact of stress caused by violence on associative learning and memory consolidation.

This work also introduces a novel behavioral testing paradigm that combines high-dimensional tracking with established cognitive tasks to improve detection sensitivity. Traditional assays like the novel object recognition test often yield inconsistent results and high false-negative rates in models of mild cognitive impairment [23,24,25]. In contrast, our integrated system captured subtle recognition impairments—such as a ≥30% reduction in novelty-directed sniffing in ASCV mice—that were undetectable by traditional metrics. Similarly, Skinner box performance revealed a 35% decrease in lever-press accuracy and a two- to three-fold increase in response latency following ASCV. This multidimensional framework significantly enhances diagnostic sensitivity for cognitive impairment models and provides a robust platform for pharmacological screening.

We also found that excitatory neurons in the DG respond to ASCV-induced cognitive impairment, and inhibiting these neurons alleviates the dysfunction. Previous work showed that DG inhibition during adolescence increases activity in hippocampal CA3/CA1 and impairs social and spatial working memory, whereas adult inhibition does not produce the same effects [26]. Because CaMKII is a broad marker for excitatory neurons, the specific DG subtypes mediating post-stress cognitive deficits remain unclear. Our study further demonstrates that inhibiting the DG in adult mice not only spares mild cognitive function but can also alter certain cognitive impairments phenotype in the presence of violent stressors.

A 2020 study demonstrated that acute inhibition of a subset of excitatory neurons after ischemic stroke prevents brain injury and improves functional outcomes [27]. This is highly consistent with the trend of our experimental results. Therefore, we believe that inhibiting CaMKII neurons in the DG before acute stress, which alters the mild cognitive impairment phenotype, does not reverse already formed cognitive dysfunction but rather prevents the emergence of the phenotype. CNO intervention interferes with the encoding of the cognitive dysfunction phenotype induced by stress, thereby preventing the corresponding phenotype from developing in the hM4Di group mice.

Several limitations should be noted. The current model did not incorporate direct neural circuit activity analysis. Although the hippocampus is central to memory consolidation [21,22] and the periaqueductal gray is involved in stress modulation [23,24], we did not evaluate specific neuronal dynamics in the DG. Molecular correlates of behavior—such as stress-responsive serum biomarkers (e.g., fibulin-1 [25]) or exosomal cargo—were also not examined. Furthermore, only male mice were used despite epidemiological evidence of higher female susceptibility to stress-related disorders [26,28]. Future studies should integrate neural circuit dynamics, molecular profiling, and sex-stratified analyses to better reflect clinical heterogeneity.

By employing a multidimensional behavioral assessment system, this study confirmed ASCV-induced spatial memory impairment and revealed the regulatory role of DG excitatory neurons in this process. It also highlighted the limitations of traditional paradigms in detecting subtle cognitive deficits. Combining 3D-resolved exploratory patterns with dynamic learning metrics provides a more sensitive framework for evaluating post-stress cognitive dysfunction. Future work combining optogenetic modulation and multiomics approaches will further elucidate the neurobiology of stress-induced cognitive decline, ultimately informing clinical diagnostics for stress-associated psychiatric disorders.

## 4. Materials and Methods

### 4.1. Animals

All animal procedures were conducted in accordance with the Institutional Animal Care Committee of Xi’an Jiaotong University and the Research Committee of Shenzhen Institutes of Advanced Technology of the Chinese Academy of Sciences. Experimental protocols complied with institutional and national guidelines for the care and use of laboratory animals. Male C57BL/6J mice (8–12 weeks old), exhibiting normal morphology and body weight, were housed under controlled environmental conditions with a 12 h light/dark cycle (lights on 08:00–20:00) and ad libitum access to food and water. All behavioral assessments were performed during the light phase to minimize circadian variation.

### 4.2. Behavioral Assessments

To reduce novelty-induced stress, mice were handled for 10 min daily over a 3-day habituation period. Conventional behavioral tests were video-recorded and analyzed using ANY-maze v7.1.

#### 4.2.1. Acute Stress Caused by Violence (ASCV) Model

An ASCV model was adapted from the chronic social defeat stress paradigm [29] and the resident-intruder paradigm to simulate aggressive encounter-induced trauma. The protocol involved three steps: (1) Induction of physiological stress: CD-1 mice were singly housed for 7 days to establish territorial behavior; (2) Subject introduction: After acclimation, C57BL/6J mice were introduced into the home cage of an aggressive CD-1 mouse; (3) Aggression phase: Experimental mice were subjected to a minimum of 10 attack episodes within 20 min.

#### 4.2.2. Novel Object Recognition Test

Spatial memory was assessed using the novel object recognition test [30]. Mice were placed in a square open field containing two identical, scent-neutral objects (A and B), positioned 5 cm from the walls. Each mouse was introduced facing away from the objects and allowed to explore for 15 min. After a 2 h retention interval, one familiar object was replaced with a novel item (A/C or B/C), and mice were reintroduced for a second 15 min session. Exploration times for each object were recorded to calculate discrimination indices. The arena and objects were cleaned with 75% ethanol between trials to remove olfactory cues.

#### 4.2.3. Y-Maze Test

Spatial working memory was assessed using a standardized Y-maze [31]. The apparatus consisted of three arms of equal length (30 × 6 × 15 cm) arranged at 120° angles. Mice explored freely for 10 min, and movements were recorded using ANY-maze. The maze was cleaned with 75% ethanol between trials.

#### 4.2.4. Skinner Box Test

Operant conditioning was evaluated using a protocol adapted from established reinforcement-based assays [32,33]. Mice were trained in Skinner boxes to associate left lever presses with a 10% sucrose solution reward under a fixed-ratio 1 (FR-1) schedule. Each 60 min training session was repeated for 5 consecutive days. Memory retention was evaluated 3 weeks after training. Behavior was recorded using the SA229 Skinner box system (SA Series, Sans Bio, Nanjing, China) and Skinner RGB Trum Pet software (SA229 Skinner Box, Version 2.0). The chamber was cleaned with 75% ethanol between trials.

### 4.3. Three-Dimensional Behavioral Atlas Analysis

High-resolution behavioral phenotyping followed established protocols from prior experimental platforms [17]. Mice were habituated to the test arena for 60 min, acclimated in the open field for 15 min, and then recorded for 15 min of spontaneous behavior. The arena was sanitized between sessions.

#### 4.3.1. Three-Dimensional Behavioral Video Acquisition

Behavior was recorded in a transparent acrylic open-field apparatus (40 × 40 × 40 cm) with a frosted white floor to enhance contrast for motion tracking. Four synchronized high-definition cameras (Intel RealSense D435, Inte, Shanghai, China) captured spontaneous behaviors from multiple angles. The arena was housed in a soundproof black chamber under diffuse white lighting to minimize external interference. Multi-view video data were acquired through a 3D motion capture system (BAYONE, Shenzhen, China).

#### 4.3.2. Three-Dimensional Behavioral Data Analysis

Cameras were calibrated prior to recording. Skeletal trajectory data from all four cameras were integrated to reconstruct body part positions across timeframes, generating average skeletal models. Video data were imported into the Behavior Atlas platform for classification and analysis. Deep Lab Cut was employed to train body part tracking across individual camera feeds, yielding 2D skeletal trajectories. Machine learning algorithms automatically identified behavioral phenotypes, with undefined behaviors subjected to cluster analysis. Three-dimensional skeletal data facilitated behavioral deconstruction and unsupervised clustering, identifying 40 distinct action modules and their frequencies.

#### 4.3.3. Three-Dimensional Behavioral Atlas Action Clustering

To improve interpretability and reduce dimensionality, the 40 initial action modules were consolidated through a multi-layered pipeline. Spatiotemporal relationships among non-locomotive behaviors were quantified by video replay analysis, motion pattern reconstruction, and principal component analysis (PCA)-based distance metrics with non-motile metrics (NM) and the dynamic time alignment kernel (DTAK) algorithm. Uniform Manifold Approximation and Projection (UMAP) was applied to further reduce high-dimensional NM features into 2D space, enhancing pattern visualization.

Unsupervised clustering of integrated 3D behavioral features, supplemented by expert annotation, identified 14 phenotypically distinct action subclasses. These were categorized into four functional clusters: locomotion, exploration, maintenance, and inactivity. Data were standardized and analyzed using Prism v7.0 for multivariate statistics, with results visualized via heatmaps and PCA scatterplots.

### 4.4. Chemogenetic Manipulation and Stereotactic Virus Injection

Viral vectors were loaded into an injection needle using a microinjection pump (RWD, Shenzhen, China). Mice were anesthetized with 1% sodium pentobarbital (Sigma-Aldrich, Shanghai, China, #P3761; 10 mL/kg, i.p.) and secured in a stereotaxic frame. The experimental group received AAV-CaMKII-hM4Di-mCherry (5 × 10^12^ particles/mL), while controls received AAV-CaMKII-mCherry (5 × 10^12^ particles/mL). The injection needle was wiped with cotton and lowered into the dentate gyrus (AP: −1.00 mm, ML: +0.45 mm, DV: −2.70 mm). Virus was delivered at 50 nL/min. After injection, the needle remained in place for 10 min before slow withdrawal. The scalp was sutured, the incision was disinfected with iodine, and mice were placed on a warming pad until fully recovered before returning to a clean cage. Chemogenetic activation of the circuit was achieved by intraperitoneal injection of CNO (1 mg/kg), with behavioral testing conducted 30 min post-injection. Viral vector details are listed in Table 1.

### 4.5. Immunofluorescence Staining

Mice were anesthetized with 10% urethane (10 mL/kg, i.p.) and transcardially perfused with 4% paraformaldehyde (PFA). Brains were postfixed overnight, cryoprotected in 30% sucrose, embedded in OCT, and sectioned at 40 μm. Sections containing the target region were selected for immunohistochemistry. After three 5 min washes in PBS to remove OCT, sections were blocked with 10% normal goat serum for 1 h at room temperature. Primary antibody, diluted in 0.1% PBST with Triton X-100, was applied overnight at 4 °C. Following three PBST washes, sections were incubated with species-appropriate secondary antibodies (Table 2) for 1 h at room temperature. After three final washes in PBST with Tween 20, nuclei were counterstained with DAPI (3 min) and rinsed in PBS (1 min). Sections were mounted and imaged using a Zeiss LSM880 confocal microscope (Zeiss, Oberkochen, Germany). Antibody details are provided in Table 2.

### 4.6. Statistical Analysis

Spatial and behavioral tracking data were extracted using ANY-maze v7.1 (USA). Normality and homogeneity of variance were evaluated using the Kolmogorov–Smirnov test, two-tailed *t*-test, or Mann–Whitney test for non-parametric data. One-way and two-way analysis of variance (ANOVA), followed by appropriate multiple comparisons, were employed for group analyses. Data are presented as mean ± standard error of the mean (SEM) or median, with statistical significance defined as *p* < 0.05. GraphPad Prism v9.0 (USA) was used for all analyses.

## 5. Conclusions

The high prevalence and low diagnosis rate of violence-related mental illnesses underscore the urgent need for diagnostic and therapeutic strategies targeting violence-induced cognitive impairment. By integrating novel and conventional approaches, this study demonstrates that acute stress caused by violence induces mild cognitive impairment in mice, that the DG is a responsive node in this process, and that inhibiting DG excitatory neuronal activity prevents the resulting dysfunction. Our findings identify a specific brain region and neuronal target for understanding violence-induced cognitive impairment and suggest new directions for preventing cognitive damage in victims of violence.

## Figures and Tables

**Table 1:** Virus List.

Virus	Catalog Number
AAV-CaMKII-hM4Di-mCherry	BC-0150 (Brain Case)
AAV-CaMKII-mCherry	BC-0028 (Brain Case)

**Table 2:** Immunofluorescence Staining Antibody List.

Antibody	Catalog Number	Dilution Ratio
anti-c-Fos (Rabbit mAb) (CST, Shanghai, China)	CST-2250S	1:200
594 (Goat anti-Rb) (Abcam, Shanghai, China)	Abcam ab150083	1:200
DAPI (Sigma-Aldrich, Shanghai, China)	D-9542	1:5000

## Data Availability

The original contributions presented in this study are included in the article and Supplementary Materials. Further inquiries can be directed to the corresponding authors.

## Supplementary Materials

The following supporting information can be downloaded at https://www.mdpi.com/article/10.3390/ijms27010226/s1.
