# Supplementary material for: CaMKII Neurons in the Dentate Gyrus Are Involved in Regulating Cognitive Impairment in Mice Induced by Stress Caused by Violence
Source: Int J Mol Sci. 2025 Dec 25;27(1):226. doi: 10.3390/ijms27010226 (PMC12785653; doi:10.3390/ijms27010226)
Supplement: Supplementary file 1 [file ijms-27-00226-s001.zip › ijms-4015670-supplementary.pdf]

Supplymentary

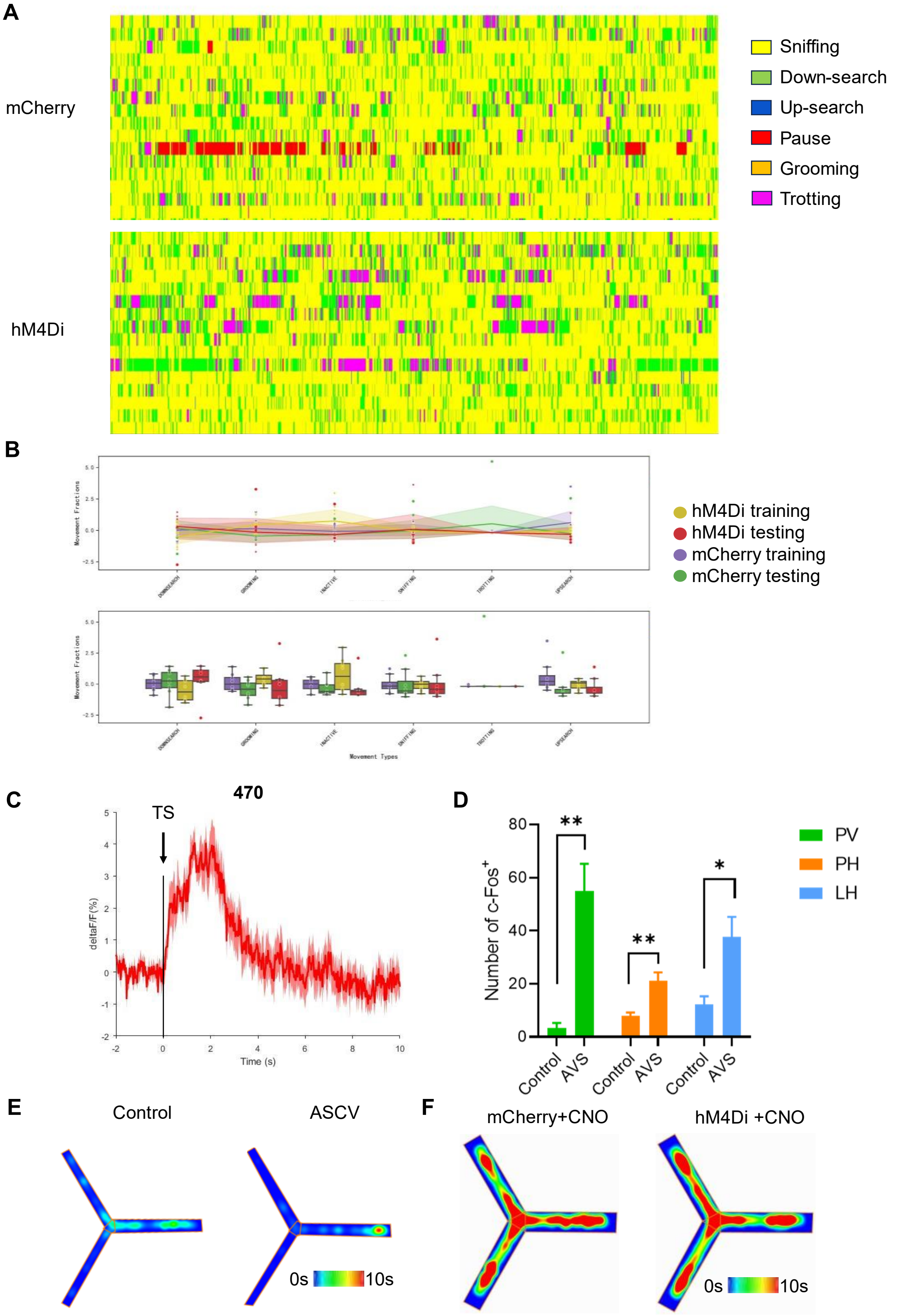

Figure S1 DG responds to negative stimuli, other brain regions with differential c-Fos expression induced by ASCV.

A. Action spectrograms of five behavioral subclasses in mCherry and hM4Di during

integrated 3D-object recognition tasks. (n=8/group)

B. Scatter plots and bar charts showing the proportion of different types of action segments relative to the total number of action segments. (n=8/group)

C. Gcamp6s response to TS: (Left) Peri-event ΔF/F trace, (Right) Response heatmap. (n=3–4/group).

D. Quantification of c-Fos expression in the PVN, LH, and PH. (unpaired t-test: PH: $t = 3.735$, $p = 0.0039$; PV: $t = 4.974$, $p = 0.0025$; LH: $t = 3.120$, $p = 0.0109$). (n=5-6/group).

E.Y-maze heatmaps for Control and ASCV groups. (n=5/group)

F. Y-maze heatmaps for mCherry and hM4Di group following CNO administration. (n=8/group)
